# Targeted Promoter Replacement Reveals That Herpes Simplex Virus Type-1 and 2 Specific VP16 Promoters Direct Distinct Rates of Entry Into the Lytic Program in Sensory Neurons *in vivo*

**DOI:** 10.3389/fmicb.2019.01624

**Published:** 2019-07-24

**Authors:** Richard L. Thompson, Nancy M. Sawtell

**Affiliations:** ^1^Department of Molecular Genetics, Biochemistry, and Microbiology, University of Cincinnati, Cincinnati, OH, United States; ^2^Department of Pediatrics, Division of Infectious Diseases, Cincinnati Children’s Hospital Medical Center, Cincinnati, OH, United States

**Keywords:** herpes simplex virus, latent program, sensory neuron, HSV-1 and HSV-2 VP16 promoter, regulation, corneal infection model, trigeminal ganglion, serotype

## Abstract

Infection and life-long residence in the human nervous system is central to herpes simplex virus (HSV) pathogenesis. Access is gained through innervating axonal projections of sensory neurons. This distinct mode of entry separates the viral genome from tegument proteins, including the potent transactivator of viral IE genes, VP16. This, in turn, promotes a balance between lytic and latent infection which underlies the ability of the virus to invade, disseminate, and set up a large reservoir of latent infections. In the mouse ocular model, TG neurons marked as either “latent” or “lytic” at 48 h postinfection indicated that these programs were selected early and were considered distinct and mutually exclusive. More recently, a temporal analysis of viral program selection revealed a default latent-like state that begins at ~18 h postinfection and in individual neurons, precedes entry into the viral lytic cycle. Studies using refined viral mutants demonstrated that transition out of this latent program depended upon the transactivation function of VP16. Pursuit of the apparent incongruity between the established leaky-late kinetics of VP16 expression with a “preimmediate-early” function led to the discovery of an unrecognized regulatory feature of the HSV-1 VP16 promoter near/downstream of its TATA box. Among three potential sites identified was a putative Egr-1/Sp1 site. Here, we report that a refined mutation of this site, while having no impact on replication in cultured cells or cornea, resulted in ~100-fold reduction in lytic infection in TG *in vivo*. Notably, the HSV-2 VP16 promoter has 13 direct tandem-repeats *upstream* of its TATA box forming multiple potential overlapping Egr-1/Sp1 sites. Thus, despite different structures, these promoters might share function in directing the preimmediate-early VP16 protein expression. To test this, the HSV-1 VP16 promoter/5′UTR was replaced by the HSV-2 VP16 promoter/5′UTR in the HSV-1 backbone. Compared to the genomically repaired isolate, the HSV-2 VP16 promoter/5′UTR (1) accelerated the transition into the lytic cycle, and enhanced (2) virulence, and (3) entry into the lytic cycle following a reactivation stressor. These gain-of-function phenotypes support the hypothesis that the VP16 promoter regulates the latent/lytic boundary in neurons and that the HSV-1 and HSV-2 promoter/5′UTRs encode distinct thresholds for this transition.

## Introduction

The US and global disease burden resulting from herpes simplex virus (HSV-1 and -2) infection includes life threatening encephalitis, blindness, devastating neonatal infection, increased risk of HIV infection, neurological disease, and a list of other disease outcomes. Life-long latent infections in sensory neurons and periodic reactivation of latent virus are central to the maintenance and propagation of these viruses, currently a worldwide endemic infection. Practical treatments to prevent transmission or eliminate the latent reservoir remain unmet goals. Greater insights into the regulatory mechanisms controlling latent and lytic program selection in the nervous system are needed to facilitate progress. Virion protein 16 (VP16) is a multifunctional viral protein expressed with leaky late kinetics and essential for virion morphogenesis. VP16, in complex with host proteins, is also a potent transactivator of the 5 HSV Immediate Early genes. In cultured cells, efficient infection by a single HSV-1 virion requires the VP16 transactivation function which is transported into the cell as part of the virion tegument (Campbell et al., 1984; Kristie and Roizman, 1987; Ace et al., 1988; Stern et al., 1989).

Most often, infection of the host with HSV starts at an external epithelial cell surface where viral replication leads to access and uptake into the nervous system *via* axons projecting from the neuronal cell body in the sensory ganglion. In the case of the sensory neuron, this single axon bifurcates in the ganglion near the cell body and projects to the CNS. Early studies in cultured neurons infected *via* axons did offer strong support for the idea that latency might be favored in neurons because the tegument protein VP16 is not efficiently transported to neuronal nuclei to initiate lytic infection (Miranda-Saksena et al., 2000; Diefenbach et al., 2008). This important finding was supported and extended by Hafezi et al. (2012) who showed that infection of cultured neurons *via* axons led to quiescent infection whereas infection on the cell body led to lytic infection, supporting early *in vivo* studies (Ecob-Prince et al., 1993). More recently, the addition of light particles containing pseudorabies virus tegument proteins disrupted quiescent infection of cultured neurons infected *via* axons, again implicating the presence or absence of tegument proteins in the switch between quiescent and lytic infection of neurons (Koyuncu et al., 2017).

*In vivo*, evidence that TG neurons marked as either “latent” or “lytic” as soon as 48 h post infection indicated that the latent or lytic program decision was being made during the acute stage of infection (Margolis et al., 1992; Sawtell and Thompson, 1992a,b; Lachmann and Efstathiou, 1997). This led to the hypothesis that the choice between latent or lytic infection occurred early during infection and was mutually exclusive and correlated with neuronal subsets (Margolis et al., 1992, 2007; Sawtell and Thompson, 1992a,b; Lachmann and Efstathiou, 1997).

More recently, a temporal analysis of program selection in TG neurons revealed that a characteristic feature of infection of the TG neuron from the periphery is an unexpected default latent state that occurs early in individual TG neurons. This default latency begins as early as 18 h post infection and precedes all lytic gene transcription by 12–14 h. Importantly, studies using a series of refined viral mutants demonstrated that the transition out of this latent program is dependent on VP16 transactivation function (Thompson et al., 2009; Sawtell and Thompson, 2016a,b). Further examination of the apparent incongruity between the established leaky late kinetics of VP16 expression with a pre-immediate early (preIE) function led to the discovery of previously unrecognized regulatory features of the VP16 promoter mapping near and downstream of the VP16 gene TATA box which directs *de novo* expression of VP16 (Sawtell and Thompson, 2016a,b). Thus *in vivo*, evidence is building that VP16 can function as a potent transactivator of viral IE genes in the sensory neuron and the regulation of this protein, either by limiting its transport into the neuron, or through its promoter regulating its expression as a pre-immediate early gene, is central in controlling the latent to lytic transition in neurons (Miranda-Saksena et al., 2000; Diefenbach et al., 2008; Thompson et al., 2009; Antinone and Smith, 2010; Thompson and Sawtell, 2010; Roizman et al., 2011; Sawtell et al., 2011; Aggarwal et al., 2012; Hafezi et al., 2012; Kim et al., 2012; Smith, 2012; Sawtell and Thompson, 2016a,b). Significantly, the regulation of expression of preIE VP16 transactivation function early during acute infection of TG is linked to downstream pathogenic outcomes, emphasizing the biological significance of this regulatory strategy.

How this context dependent expression strategy of VP16 is designed is not yet completely understood. Mutation of three putative transcription factor (TF) binding sequences near and downstream of the VP16 gene TATA box greatly decreases lytic cycle entry in TG neurons infected from the eye (Sawtell and Thompson, 2016a,b). Among these is a canonical overlapping Egr-1/Sp1 site. It is likely that the regulation of this gene in neurons *in vivo* is quite complex as has been seen in other systems. We refer to this as an “Egr-1/Sp1” site, but it should be recognized that many TFs could potentially bind to this region. In addition, interpretation of mutations in the 5′UTR is complicated because this region can influence transcriptional regulation and also protein production through additional mechanisms (Leppek et al., 2018). Indeed, analysis of how gene promoters function in the context of a living animal has proven to be one of the more intractable problems in molecular genetics. Of interest, the sequence of the HSV-2 VP16 promoter/5′UTR has a strikingly different architecture compared to that of HSV-1 and the 5′UTR Egr-1/Sp1 site is not apparent. There are, however, 13 direct tandem repeats of a sequence that can form multiple strong putative Egr-1/Sp1 sites upstream of the HSV-2 TATA box. The presence of these similar direct tandem repeats suggested that despite their very different structure, these divergent promoters may function similarly in directing *de novo* VP16 protein expression in neurons. If the 13 tandem sites do increase the transition from default latent to lytic infection in sensory neurons *in vivo*, replacing the HSV-1 VP16 promoter with the HSV-2 VP16 promoter would be expected to alter the pathobiological properties of HSV-1 in the absence of all other HSV-2 adaptations and should be measurable. Importantly, *in vivo* phenotypic differences between the promoters could then be exploited to provide insight into key features of structure function relationship encoded in this regulatory region.

To begin to test this hypothesis, we first mutated the HSV-1 VP16 promoter disrupting only the putative Egr-1/Sp1 site within the 5′UTR. The *in vivo* phenotype of this mutant paralleled that of the triple site mutant described previously (Sawtell and Thompson, 2016a,b), establishing the importance of this sequence to the regulation of *de novo* VP16 expression. We next generated HSV-1 mutants in which the HSV-1 VP16 promoter and 5′UTR is replaced by the HSV-2 VP16 promoter and 5′UTR. All other viral promoters and all viral ORFs remain HSV type-1 in this targeted VP16 promoter replacement mutant. Compared to the genomically repaired mutant, in the context of the HSV-1 genome, the HSV-2 VP16 promoter/5′UTR (1) accelerates the transition from default latency into the lytic cycle, and enhances (2) virulence, and (3) entry into the lytic cycle following a reactivation inducing stressor by fourfold. These unusual gain of function phenotypes further support the hypothesis that the VP16 promoter regulates the latent/lytic boundary in neurons and that the HSV-1 and HSV-2 promoter/5′UTRs encode distinct thresholds for this transition.

## Materials and Methods

### Ethics Statement

All procedures in mice were performed as approved by the Children’s Hospital Institutional Animal Care and Use Committee (protocol# IACUC2017-0081 and were in compliance with the *Guide for the Care and Use of Laboratory Animals*. Animals were housed in American Association for Laboratory Animal Care-approved quarters.

### Viral Strains/Mutants and Stock Production

An elite stock of HSV-1 strain 17syn+ (originally obtained from John H. Subak-Sharpe at the MRC Virology Unit in Glasgow, Scotland) was generated in 1980 and employed to make early passage working stocks. The wt and mutant viruses employed in this study were generated in rabbit skin cell (RSC) monolayers (RSC originally obtained from Bernard Roizman, University of Chicago) and the viral titers were determined by serial-dilution plaque assay (Thompson et al., 1983; Sawtell and Thompson, 1992a,b).

### Construction of Mutated VP16 Promoters and Viral Mutants

#### Mutation of the Putative HSV-1 Overlapping Egr-1/Sp1 Site

**Figure 1:**
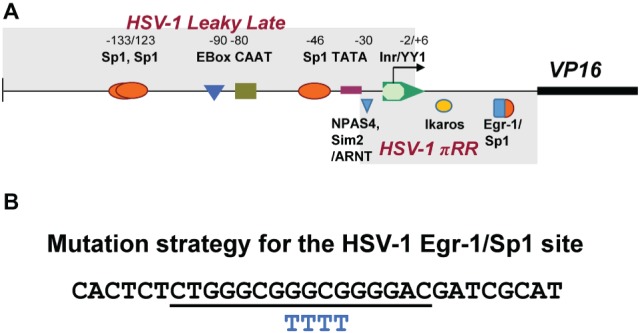
Schematic representation of transcription factor (TF)-binding sites in HSV-1 VP16 promoter. **(A)** Shown above the line are the names of TF-binding sites identified as important for VP16 promoter strength Wagner and colleagues in transient assays (Lieu and Wagner, 2000). Below the line are high-scoring TF sites identified as potentially important for *de novo* VP16 expression (boxed and labeled as HSV-1πRR) that were simultaneously mutated resulting in failure to initiate lytic gene expression in sensory neurons *in vivo* (Sawtell and Thompson, 2016a,b). **(B)** Shown is the sequence of a 30mer oligonucleotide that contains a canonical overlapping Egr-1/Sp1 site (underlined) in the HSV-1 VP16 gene 5′UTR. Below this are shown the four bases that were changed to “T” residues to mutate the TF sites (BP 105,134–105,137).

Four bases in the core matrix of the canonical overlapping Egr-1/Sp1 site in the region of the 5′ UTR of the VP16 mRNA (BP 105,134 to 105,137 on the 17syn+ genome were mutated to “T” residues (the construct was purchased from Blue heron Biotec) (see Figure 1B). All restriction enzyme sites and base pair numbering are referred to as the corresponding positions in the published HSV-1 sequence of strain 17syn+ (McGeoch et al., 1988; Perry and McGeoch, 1988) as currently present in Genbank (JN555585). This mutation is predicted to inhibit binding of both Egr-1 and Sp1 to the site as determined with the analysis suite Cis-BP (Weirauch et al., 2014). The construct was employed to drive the firefly luciferase gene (Clontech) and was also cloned in place of the native VP16 promoter in front of the VP16 ORF, 3′UTR, and polyA+ sequences and flanked by ~500 bp of homologous sequences to facilitate recombination into the viral genome as previously detailed (Thompson et al., 2009; Sawtell and Thompson, 2016a,b). The mutation construct was recombined into a previously published mutant of strain 17syn+ modified with an insertion of a GFP gene driven by the beta actin promoter and terminated with the SV40 poly adenylation sequences (SVA+) (Thompson et al., 2009), which was employed for color selection of plaques as previously described (Thompson et al., 2009; Sawtell and Thompson, 2016a,b). Six independently derived mutants were selected, plaque purified by three rounds of limiting dilution and sequences of the insertion were confirmed by PCR followed by Sanger sequencing (Genewiz). General genomic structures were examined by RFLP analysis as previously described in detail (Sawtell and Thompson, 2014). Three mutants (named 17VP16pEgr-1/Sp1-1, 3, or 5) were characterized *in vitro* and *in vivo* as described in the text. One isolate was genomically restored to wt sequence (using the wt HSV-1 VP16 promoter +5′UTRsequences) to generate the control virus named 17VP16pEgr-1/Sp1-R. Virus plaque purification and characterization were as described above.

#### Targeted Replacement of the HSV-1 VP16 Promoter and 5′UTR Sequences with Those of HSV-2

**Figure 2:**
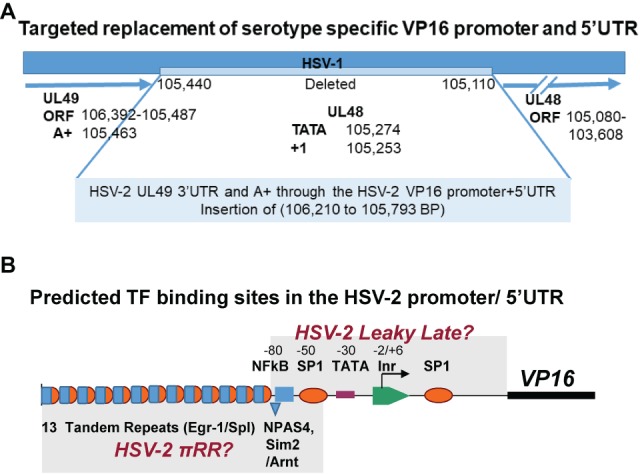
Design of the targeted replacement of the HSV-1 VP16 promoter and 5′UTR with that of HSV-2, mutant 17LpLz/VP16p^2^. **(A)** Note that the VP16 (UL48) gene runs in the opposite direction of the base pair numbers of the HSV genome in the prototypical genome arrangement. The HSV-1 promoter and UTR sequences (105,440 to 104,110 bp) were deleted and replaced with leaving the HSV-2 VP16 promoter and 5′UTR (106,210 to 105,793 bp) as detailed in Section “Materials and Methods.” The VP16 ORF and 3′UTR remained entirely HSV-1 sequences. This mutant is designated 17LpLz/VP16p^2^. **(B)** A schematic representation of the HSV-2 VP16 promoter and 5′UTR is shown. Putative binding sites for TFs that are thought to regulate HSV leaky late promoters (Lieu and Wagner, 2000) were identified *in silico* (see Section “Materials and Methods”) and are indicated above the line. Two regions containing TF-binding sites similar to those implicated in the *de novo* synthesis of HSV-1 VP16 (Sawtell and Thompson, 2016a,b) are indicated below the line including tandem repeats of putative overlapping Egr-1/Sp1 sites and a potential NPAS4 (positive TF) or Sim2 (negative TF)/Arnt heterodimer binding site.

The HSV-1 VP16 promoter and 5′ UTR sequences (BP 105,440 to 105,110) were replaced with the HSV-2 VP16 promoter/5′UTR (BP 106,210 to 105,793) as found in Genbank Accession NC_001798 (Figure 2A). To help insure that all important HSV-2 VP16 regulatory sequences were included, the fragment extends 63 bp 5′ of the HSV-2 UL49 polyadenylation site (UL49 is upstream of UL48, the VP16 gene) through the HSV-2 VP16 promoter and includes all but 28 bp of the HSV-2 VP16 5′UTR, followed by the corresponding 27 bp of the HSV-1 VP16 5′UTR to preserve the HSV-1 gene Kozak consensus translation start site (Kozak, 1987). This short sequence has 67% identity in the two simplex viruses. These sequences were employed to drive firefly luciferase as above so that the relative strengths of the HSV-1 and HSV-2 regulatory regions could be compared in transient assays (see Figure 2A). Targeted replacement mutant viruses in which the HSV-1 VP16 regulatory sequences were replaced with these type-2 sequences were made as follows. The GFP cassette described above was place in front of the type-2 sequences and in the same orientation as the VP16 gene to provide color selection for plaques and flanked by ~500 bp of relevant HSV-1 sequences on the 5′ and 3′ ends of the construct to permit efficient recombination with viral genomic sequences. The mutagenesis construct was recombined into the mutant 17LATpLacZ [which marks the latent transcription program (Thompson et al., 2009; Sawtell and Thompson, 2016a,b)] to generate mutants 17LpLz/VP16p^2^. Note that all HSV-1 viral genes including all ORFs remain intact in these mutants with the exception of exchanging the HSV-1 VP16 promoter and most of its 5′UTR with that of the HSV-2 VP16 promoter and most of its 5′UTR.

As above, all recombination crossover sites were confirmed by PCR and sequencing. The presence of the 13 tandem direct repeats of the canonical Egr-1/Sp1 site present in the type-2 promoter was confirmed by sequence and by high-resolution RFLP analysis as previously detailed (Sawtell and Thompson, 2014). Six independent derived promoter mutants were isolated and two of these (named 17LpLz/VP16p^2^-5 and 6) were further characterized *in vitro* and *in vivo* as described in the text. The genome of 17LpLz/VP16p^2^-5 was rescued back to wildtype (wt) to generate the control virus 17LpLz/VP16p^2^-R in which the HSV-1 VP16 regulatory sequences were restored.

### Luciferase Assays

**Figure 3:**
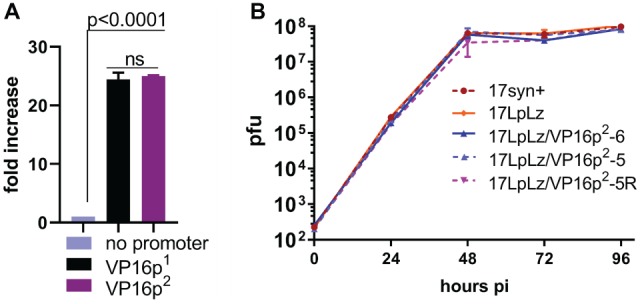
Promoter function and *in vitro* multi-step viral replication kinetics. **(A)** A plasmid containing the wt HSV-1 VP16 gene (designated here as VP16p^1^), the same plasmid with the VP16 promoter deleted (designated as no promoter), and the plasmid generated to produce the HSV-2 VP16 regulatory sequence targeted replacement (designated here as VP16p^2^, see Figure 4A) were employed in transient dual luciferase assays with a previously reported ICP0 promoter/Luciferase target plasmid (Sawtell and Thompson, 2016a,b) to test their ability to express VP16 and activate the ICP0 promoter as detailed in Section “Materials and Methods.” The “no promoter” level of luciferase detected was set to one. Each bar represents the results of six transfection experiments. **(B)** Multi-step replication kinetics was performed in RSC. Each point is the average ± S.D. of three independently infected cultures harvested and titrated at the indicated times pi. Wild-type HSV-1 strain 17syn+ is the parent strain of 17LATpLacZ (here indicated as 17LpLz) which expresses beta-galactosidase from the LAT promoter as previously described (Sawtell and Thompson, 2016a,b). 17LATLpLacZ is the parent strain of the mutants generated for this study (17LpLzVP16p^2^-5 and 6) and the genomically restored isolate 17LpLzVP16p^2^-5R.

Dual-Glo luciferase assays (Promega) were employed in co-transfection assays according to the manufacturers’ protocols. To assay for promoter strengths, the constructs in which firefly luciferase was driven by wt or mutant VP16 promoters as well as a cytomegalovirus immediate early gene promoter (CMVIE) driven luciferase construct (Promega) as a positive control, were co-transfected with the pRL-TK renilla expression plasmid (Promega) or no promoter vector. The HSV-2 regulatory sequences were slightly stronger in this assay (≤2-fold higher). To assay for VP16 function, the VP16 constructs employed to make the mutants described above were co-transfected with an ICP0 promoter (124,818 to 124,109 BP) firefly luciferase construct (Sawtell and Thompson, 2016a,b). Transfection efficiency was determined and normalized by including the relevant renilla or firefly luciferase expression plasmids (Figure 3A).

### Electrophoretic Mobility Shift Assays

Kits for the detection of Sp1 and Egr-1 were purchased from Signosis. The commercial oligonucleotide target for Egr-1 and Sp1 are shown along with their position weighted matrix PWM LogOdds scores determined with cis-BP (Weirauch et al., 2014) Egr-1 ATCCAGCGGGGGCAGCGGGGGCGA 15.6; Sp1 ATTCGATCGGGGCGGGGCGACTGAT 16.5. The 30mer HSV-1 oligonucleotide shown in Figure 1B (Invitrogen) scored 11.1 and 12.9 for Egr-1 and Sp1, respectively. The oligonucleotides were end labeled with T4 polynucleotide kinase (NEB) and γ-32p-ATP (Perkin Elmer) and hybridized to commercial recombinant Sp1, Egr-1, or HeLa cell nuclear extract and electrophoresed according to the manufacturers’ protocols (Signosis and Sigma Aldrich). Blots were developed and analyzed on a Storm phosphoimager and quantified with GelQuantNet software. The repetitive elements in the HSV-2 sequence are GGGGCGGGAGGGGCGGGAGGGGCGGGAGGGGCGGGAGGGGCGGGAGGGGCGGGAGGGGCGGGAGGGGCGGGAGGGGCGGGAGGGGCGGGAGGGGCGGGAGGGGCGGGAGGGGCGGG, and their PWM LogOdds scores are 11.1 (Egr-1) and 23.1 (Sp-1) at multiple sites.

### Antibodies and Immunohistochemistry

Following removal, TG was placed in 0.5% formaldehyde (prepared from paraformaldehyde) for 2 h, rinsed in phosphate buffered saline, and incubated in x-gal buffer containing x-gal for 6 h as detailed previously (Sawtell and Thompson, 2014). Following rinsing, ganglia were post fixed in methanol containing 5% DMSO for 12 h. Hydrogen peroxide was then added to a final concentration of 10% and incubated for 1 h. Ganglia were rinsed twice in methanol and stored at −80°C for a minimum of 24 h. Viral proteins were detected in whole ganglia as described previously (Sawtell, 2003). Primary antibodies used included rabbit anti-HSV (Accurate, AXL237) at 1:3,000, or rabbit anti-VP16 antibody, 1:1,000 (Clonetech) followed by the secondary antibody, HRP-labeled goat anti-rabbit (Vector) at 1:500. Color development was achieved by incubating ganglia in a 0.1 M Tris (pH 8.2) solution containing 250 μg of diaminobenzidine (Aldrich)/ml and 0.004% H_2_O_2_ for approximately 5 min. Ganglia were rinsed in distilled water and cleared in glycerol to aid in visualization of the HSV protein positive neurons. These methods and the dilutions and characterizations of antibodies utilized have been detailed extensively in previous reports (Sawtell, 2003; Thompson et al., 2003, 2009; Thompson and Sawtell, 2006; Sawtell et al., 2011).

### Animals

Male, outbred, Swiss Webster mice (22–25 g in weight) were obtained from Envigo and used throughout this study. Age-matched Swiss Webster female mice (Envigo) were used in a subset of experiments as indicated.

### Inoculation of Mice

Prior to inoculation, mice were anesthetized by intraperitoneal injection of sodium pentobarbital (50 mg/kg of body weight). A 10 μl drop containing ~1–2 × 10^5^ pfu of virus was placed onto each scarified cornea.

### Pathobiological Characterization of Viral Mutants

#### Replication *in vivo*

Mice infected as above were euthanized at the indicated times post infection and tissues from a minimum three mice from each inoculation group were individually assayed for virus using a standard plaque assay. Tissue homogenates were plated in serial 10-fold dilutions on RSC monolayers, incubated for 2 h, and subsequently rinsed and overlaid with 1% carboxymethyl cellulose in minimal essential media as previously detailed (Sawtell and Thompson, 1992a,b).

### Quantification of Viral Genomes by Real-Time PCR Assay

Isolation and quantification of total DNA from TG, using the PicoGreen double-stranded DNA quantification kit according to the manufacturer’s instructions and with provided DNA standards (Molecular Probes), and quantification of total viral genomes was performed essentially as detailed previously except that the PicoGreen based real time PCR assay (Roche) was performed in a Roche 480 II instrument in a 96-well format and analyzed with the Roche 480 II LightCycler software (Sawtell et al., 2006).

### *In vivo* Stress

Transition into the lytic cycle in the ganglia of mice *in vivo* was induced using hyperthermic stress (HS) (Sawtell and Thompson, 1992a,b). At 20 h post-induction, TG was harvested and assayed for viral protein expression as detailed previously (Sawtell, 2003).

### Photomicrographs

Photomicrographs were obtained using an Olympus BX40 microscope outfitted with a Zeiss axiocam HRc camera and axiovision software. Cleared TG was viewed and marked cells populations were counted by two independent investigators.

### Statistical Analysis

Statistical analyses were performed using GraphPad Prism software (GraphPad Software, San Diego, CA). *p* < 0.05 is considered significant.

## Results

### The Egr-1/Sp1 Site in the 5′UTR of the HSV-1 VP16 Regulatory Region Can Bind Both Egr-1 and Sp1

**Figure 4:**
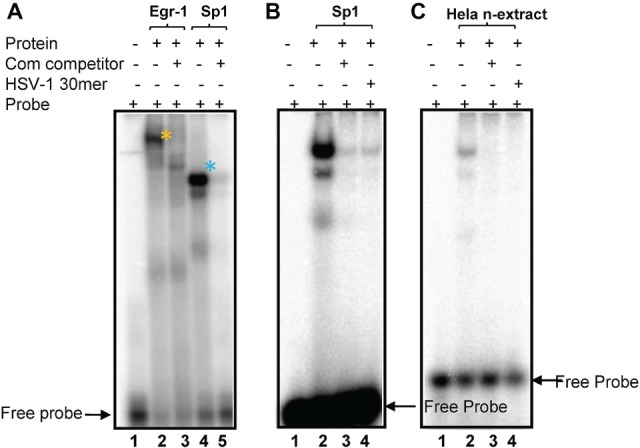
Recombinant Sp1 and Egr-1 can bind to the putative Egr-1/Sp1 site. **(A)** The HSV-1 30 bp oligonucleotide shown in Figure 1A was end labeled, incubated with recombinant Egr-1 or Sp1 protein, electrophoresed and developed on a PhosphoImager as detailed in Methods. Lanes: (1) no protein; (2) rEgr-1; (3) rEgr-1 + 40× cold probe; (4) rSp1; (5) rEgr-1 + 40× cold probe. Arrows indicated free probe (FP). Shifted probe bands are denoted with *. **(B)**. Labeled commercial probes were incubated rSp1 probe Lanes: (1) no protein; (2) rSp1 + probe. (3) +40× cold HSV-1 30mer; (4) + 40× cold probe. **(C)** Labeled HSV-1 30mer was incubated with HeLa nuclear extract as described in Section “Materials and Methods.” Lanes: (1) no nuclear extract; (2) + extract; (3) + extract +40× cold HSV-1 30mer; (4) +extract +40× commercial Sp1 probe.

Figure 1A depicts the region proposed by Lieu and Wagner to regulate leaky late expression of VP16 determined using mutated promoter analyses in transient assays (Lieu and Wagner, 2000). Mutations in these sites do not affect pre-immediate early *de novo* expression in neurons. However, simultaneous mutation of three potential TF sites identified *in silico* (labeled HSV-1πRR, Figure 1A) greatly reduced the capacity of HSV-1 to enter lytic infection in sensory neurons *in vivo*, which requires *de novo* pre-immediate early (πRR) VP16 expression (Sawtell and Thompson, 2016a,b). The sequence of the HSV-1 canonical Egr-1/Sp1 site present in the VP16 promoter 5′UTR region is shown in Figure 1B. The overlapping TF binding sites are underlined. To test whether Egr-1 and/or Sp1 could bind to this site, the HSV-1 30mer sequence shown (Figure 1B) was employed in electrophoretic mobility shift assays with recombinant Egr-1 and Sp1 proteins and HeLa cell nuclear extracts as described in Section “Materials and Methods.” Both recombinant Egr-1 and Sp1 proteins bound to the fragment with affinities similar to those found with commercial oligonucleotides for individual Egr-1 and Sp1 sites, and the HSV-1 30mer effectively competed with the canonical commercial oligos. Likewise, Sp1 present in the HeLa cell nuclear extract bound to the HSV-1 sequence efficiently and both the commercial oligonucleotide and the HSV-1 30mer competed for binding with similar efficiencies. Egr-1 was not detected in the commercial HeLa extract (Figure 4). We conclude that the 30mer sequence from the HSV-1 VP16πRR regulatory region is capable of binding these TFs with efficiencies similar to the binding of the TFs to the commercial oligonucleotides. We note that other TFs (for example diverse Krupple-like factors, etc.) would also be expected to bind to these oligonucleotides.

### Generation and Characterization of HSV-1 VP16 Promoter Mutant Constructs and Mutant Viruses

In order to test the ability of the wt promoter and the mutant promoter in which the four bases were mutated to “T” residues (Figure 1B) [predicted to eliminate binding of both Egr-1 and Sp1 (Weirauch et al., 2014)] to function as promoters in RSC in transient assays, two experiments with eight transfections each were performed using dual luciferase assays as described in Section “Materials and Methods.” Both the wt and mutant promoters functioned equivalently. The second experiment included the CMV immediate early (IE) promoter for comparison, which was about eight-fold more efficient than the VP16 promoters. No differences were detected between the wt HSV-1 VP16 and mutated putative Egr-1/Sp1 site promoters.

**Figure 5:**
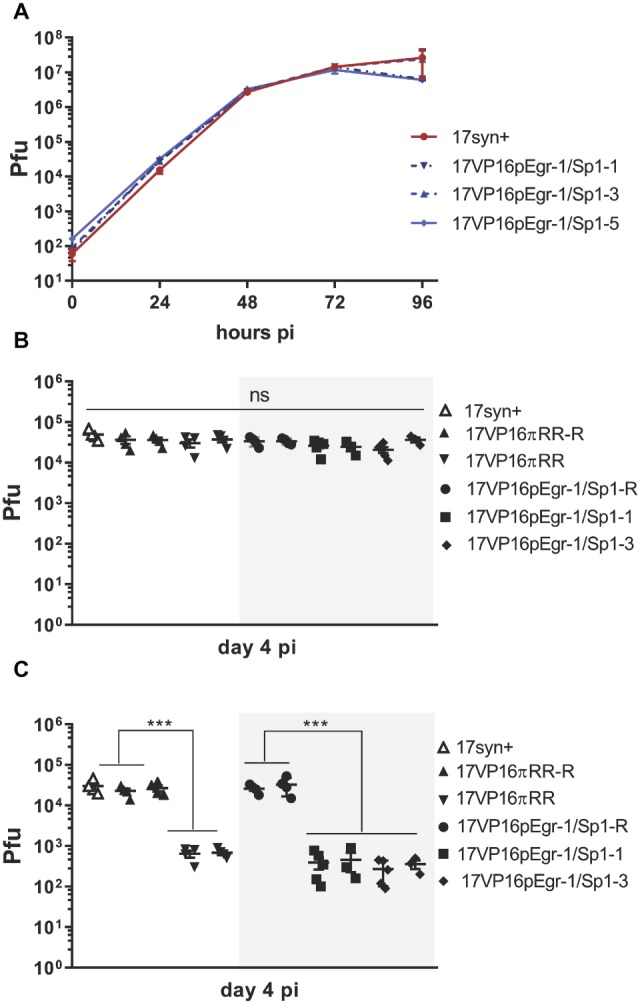
*In vitro* **(A)** and *in vivo* **(B,C)** replication of 17VP16pEgr1/Sp1, its rescue, and wild-type parental strain 17syn+. **(A)**. Multistep *in vitro* replication kinetics of parental strain 17syn+ and three independent isolates of mutant 17VP16pEgr1/Sp1. Shown is one of two biological replicates. These mutants contain a refined 4-bp mutation in the putative Egr-1/Sp1 site at position 105,134 to 105,137 in the HSV-1 VP16 promoter/5′UTR sequence (see Figure 1B). **(B)** *In vivo* replication in eyes. Groups of 4–5 mice were inoculated on scarified corneas with 2 × 10^5^ pfu of each of the 17VP16pEgr1/Sp1 and rescued viruses as indicated. In addition, groups of mice were infected with 2 × 10^5^ pfu 17VP16πRR or its rescue 17VP16πRR-R. On day 4 pi, eyes and TG were harvested and infectious viral titers quantified by standard plaque assay. Replication on the eye **(B)** is not influenced by this mutation in the Egr-1/Sp1 site in the 5′UTR. By contrast, this Egr-1/Sp1 mutation results in a significant reduction in viral titers in the TG. This effect was also observed for the triple TF 5′UTR mutant, 17VP16πRR which was reported previously (Sawtell and Thompson, 2016a,b) **(C)** Shown are results from two independent biological replicates. ***p ≤ 0.001.

We next tested the role of the putative Egr-1/Sp1 site in the regulation of VP16 in the context of the viral genome. Three independently derived viral mutants named 17VP16pEgr-1/Sp1- 1, -3, and -5, were generated, as was a genomically restored variant of one isolate (17VP16pEgr-1/Sp1-R). Procedures for plaque color selection, plaque purification, confirmation of genomic structures, and DNA sequences across the sites of insertion are detailed in Section “Materials and Methods.” No differences in replication were detected between either the independent mutant isolates, wt, or restored mutant under multi-step replication kinetic conditions in RSC (Figure 5A). Thus the mutations did not alter viral replication competency in RSC cultures (Figure 5A). Note that we previously reported that 17VP16πRR, a mutant in which three TF sites are mutated (Figure 1A; Sawtell and Thompson, 2016a,b) also replicates like wt in this assay.

Isolates 17VP16pEgr-1/Sp1-1 and -3, as well as the parental strain and the genomically restored mutant (17VP16pEgr-1/Sp1-R) were compared to the previously published 17VP16πRR mutant and its rescuant for their ability to replicate *in vivo* in the mouse cornea model. On day 4 pi, replication in the eyes was not different between any of the viruses tested, including the wt, rescuant viruses, 17VP16pEgr-1/Sp1, and the 17VP16πRR mutant viruses (Figure 5B). By contrast, while the genomically restored isolates replicated like the parental wt strain 17syn+ in the TG, the promoter mutants were severely replication impaired at this site (Figure 5C). The impaired replication in the TG is not a result of a transport deficiency, as the number of viral genomes detected in the TG at 20 hpi was not different among the groups, *p* = 0.77, ANOVA (Table 1). Combined with previous published analyses (Thompson et al., 2009; Sawtell and Thompson, 2016a,b), these findings support the hypothesis that the mutated sequence (referred to as a mutated putative Egr-1/Sp1 site) in the HSV-1 VP16 5′UTR plays a role in initiating the transition into the lytic cycle in TG neurons infected *via* axons. However, the recognized complexities of interpreting mutations in the 5′UTR region (Leppek et al., 2018) prompted an additional approach to strengthen and extend this analysis.

**Table 1:** Analysis of viral genome copies in TG at 20 h pi.

17VP16pEgr-1/Sp1	17VP16pEgr-1/Sp1-R	17VP16πRR	17VP16πRR-R	ANOVA
8,337 ± 3,412	7,333 ± 1,528	10,367 ± 5,771	7,877 ± 2,846	*p* = 0.77

*Viral genome copies/TG pair at 20 hpi n = 3 mice/group*.

### Targeted Replacement of VP16p^1^/5′UTR^1^ With VP16p^2^/5′UTR^2^ in HSV-1 Strain 17LATpLacZ

#### Generation of 17LpLz/VP16p^2^ and Rescuant, 17LpLz/VP16p^2^-R

The HSV-1 VP16 promoter is hereafter referred to as VP16p^1^, and the HSV-2 VP16 promoter is designated VP16p^2^. Unless otherwise stated, these abbreviations refer to the promoter and 5′UTR sequences. Our strategy was to exploit the HSV-2 VP16 promoter as a second natural regulator of VP16 and generate an HSV-1 mutant in which the VP16p^1^/5′UTR^1^ (105,531–105,060 bp) was replaced with that of VP16p^2^/5′UTR^2^ (106,222–105,793 bp) in the background of 17LATpLacZ (Sawtell and Thompson, 2016a,b). The parental mutant 17LATpLacZ is wt in all regards except for the insertion of the *E. coli* LacZ gene driven by the basal latency associated transcript promoter (LATp) in the intergenic region between gJ (US5) and gD (US6). As reported (Thompson et al., 2009; Sawtell and Thompson, 2016a,b), an active LATp drives expression of the bacterial beta galactosidase enzyme (B-gal) which can be detected histochemically, thus marking neurons expressing the latent transcriptional program. This feature allows analysis of viral transcriptional program utilization (latent vs. lytic) in individual TG neurons over time following corneal infection. During long term latency, this promoter marks a subset of latently infected neurons. At the early times pi we examine here, this may also be the case, however, further studies are required to determine this.

To help ensure that all relevant upstream UL48 gene regulatory sequences were captured, part of the 3′UTR of the HSV-2 UL49 gene was included, as were sequences extending through most of the 5′UTR of the HSV-2 UL48 gene (Figure 2A). A short 27 bp region immediately preceding the VP16 ORF AUG codon (Lieu and Wagner, 2000) was left intact to preserve the HSV-1 UL48 Kozak consensus sequences (Kozak, 1987) to minimize effects on translation initiation efficiency. Read through transcription would be inhibited by the three separate poly A+ signals located upstream of the 5′ end of the HSV-2 VP16 promoter sequences (detailed in Methods) (Figure 2A) The position of predicted TF-binding sites in the HSV-2 VP16 regulatory region is shown in Figure 2B. The architecture of the HSV-2 promoter is markedly different than that of HSV-1. Notably there are 13 direct tandem repeats of a sequence that forms multiple high scoring overlapping Egr-1/Sp1 sites located upstream of the VP16 gene TATA box, but a high scoring overlapping site located downstream in the 5′UTR as seen in HSV-1 is not evident. Similarly, an NPAS4 or Sim2/Arnt heterodimer site similar to a site known to confer rapid reciprocal regulation to the neuronal Drebrin gene (Woods and Whitelaw, 2002; Ooe et al., 2004) is found upstream of the HSV-2 TATA box, whereas a similar site previously implicated in the *de novo* expression of HSV-1 is located downstream of the TATA box (Sawtell and Thompson, 2016a,b). This suggests the HSV-2 pre-immediate early regulatory sequences might reside in the more distal region of the HSV-2 promoter (Figure 2A), but this remains to be determined.

Prior to construction of the HSV-2 targeted replacement VP16 promoter mutants, the mutagenesis and genomically wt HSV-1 constructs were tested in transient assays for their ability to express VP16 and to transactivate the immediate early ICP0 promoter driving luciferase as previously described (Sawtell and Thompson, 2016a,b) and detailed in Section “Materials and Methods.” No difference was seen between the HSV-1 and HSV-2 VP16 promoters in this assay (Figure 3A). Two independently derived mutants (named 17LpLz/VP16p^2^-5 and -6) were generated by homologous recombination, plaque purified, and characterized as detailed in Section “Materials and Methods,” including sequencing of crossover sites and *in vitro* replication kinetics in RSC (Figure 3B). 17LpLz/VP16p^2^-5 was restored to the wt HSV-1 genomic sequence and named 17LpLz/VP16p^2^-R, which was plaque purified and analyzed as above.

### *In vivo* Replication Kinetics

**Figure 6:**
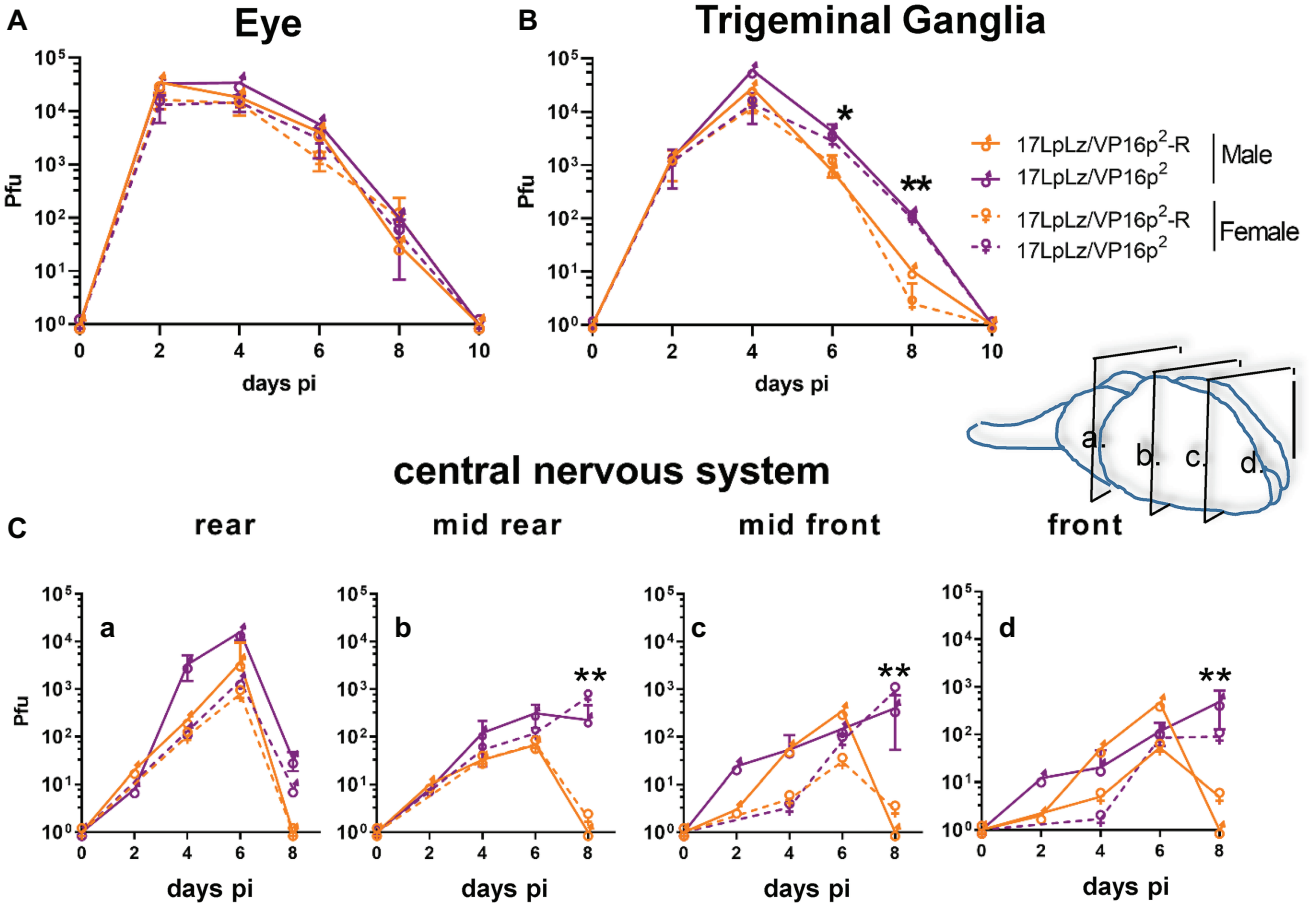
*In vivo* replication kinetics. Shown are infectious viral titers in eyes **(A)**, trigeminal ganglia **(B)**, and brains **(C)** harvested from male and female mice infected with 17LpLz/VP16p^2^ and the rescue of this mutant, 17LpLz/VP16p^2^-R. Three mice from each group were harvested at each time point indicated and infectious viral titers were quantified in tissue homogenates (see Section “Materials and Methods”). Brains were divided coronally into four pieces, labeled a–d from brainstem to front of brain as indicated in the diagram. * and ** indicate *p* = 0.01 < 0.05 and *p* = 0.001 < 0.01, respectively (unpaired, two-tailed *t* test).

**Figure 7:**
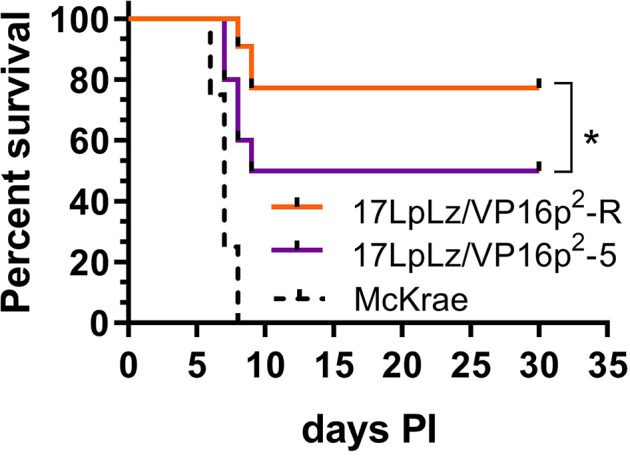
Survival curve. Shown is percent survival following infection with 2 × 10^5^ pfu of either 17LpLz/VP16p^2^ or 17LpLz/VP16p^2^-R in male Swiss Webster mice. Survival of mice infected with HSV-1 strain McKrae is shown for comparison, emphasizing that the modest increase in virulence of VP16p^2^ is well below the virulence of this naturally occurring HSV-1 strain. Data shown are compiled from two independent experiments *n* = 10 mice per group infected with either 17LpLz/VP16p^2^ or 17LpLz/VP16p^2^-R (*p* = 0.039 Log-rank Mantel-Cox test). HSV-1 strain McKrae was evaluated at the same inoculation titer in a single experiment of four mice. *p ≤ 0.05.

Analysis of replication *in vivo* in both male and female mice revealed slightly higher titers in the eyes at later times in mice infected with the 17LpLz/VP16p^2^ promoter mutant (Figure 6A), but these differences did not reach significance. In TG, only differences at later times, days 6 and 8 pi, achieved significance with 17LpLz/VP16p^2^ yielding higher titers compared to the restored isolate (Figure 6B). In the central nervous system (CNS), 17LpLz/VP16p^2^ consistently replicated to higher titers and for prolonged periods of time compared to 17LpLz/VP16p^2^-R. The absolute titers were low in the mid to front areas of the brain, but viral replication in these parts of the CNS continued past day 8 pi, when HSV-1 is typically cleared from the brain (Figure 6C; Thompson and Sawtell, 2000; Thompson et al., 2009; Sawtell and Thompson, 2016a,b). The increased neuroinvasiveness and replication in the CNS were reflected in the increased virulence of the isolates. In groups of male Swiss Webster mice infected with a total of 2 × 10^5^ pfu of 17LpLz/VP16p^2^ or 17LpLz/VP16p^2^-R, significantly more deaths occurred in the 17LpLz/VP16p^2^ group, *p* = 0.039 Log-rank Mantel-Cox test (Figure 7). However, as also shown (Figure 7), virulence was well below that of HSV-1 strain Mckrae so these viruses fall within the normal range of virulence displayed by HSV-1 strains.

### Characterization of Early Infection in TG Neurons Following Corneal Inoculation With 17LpLz/VP16p^2^: Comparison with the Rescue, 17LpLz/VP16p^2^-R

The measurable increases in replication in the TG and virulence are consistent with an HSV-2 VP16 promoter-driven shift in the early balance in the TG neuron between the latent and lytic programs (Sawtell and Thompson, 2016a,b). In order to examine this directly, we utilized the dual marker *in situ* approach with these mutants, determining the timing and number of neurons expressing LATp activity and viral protein expression to address the following questions: does the VP16p^2^/5′UTR^2^ (1) support early default latency, (2) alter its timing or stability (i.e., transition into the lytic cycle), and/or (3) alter stress induced lytic cycle entry?

### HSV-2 VP16 Promoter Mutant Enhances the Transition from the Early Default Latent State

**Figure 8:**
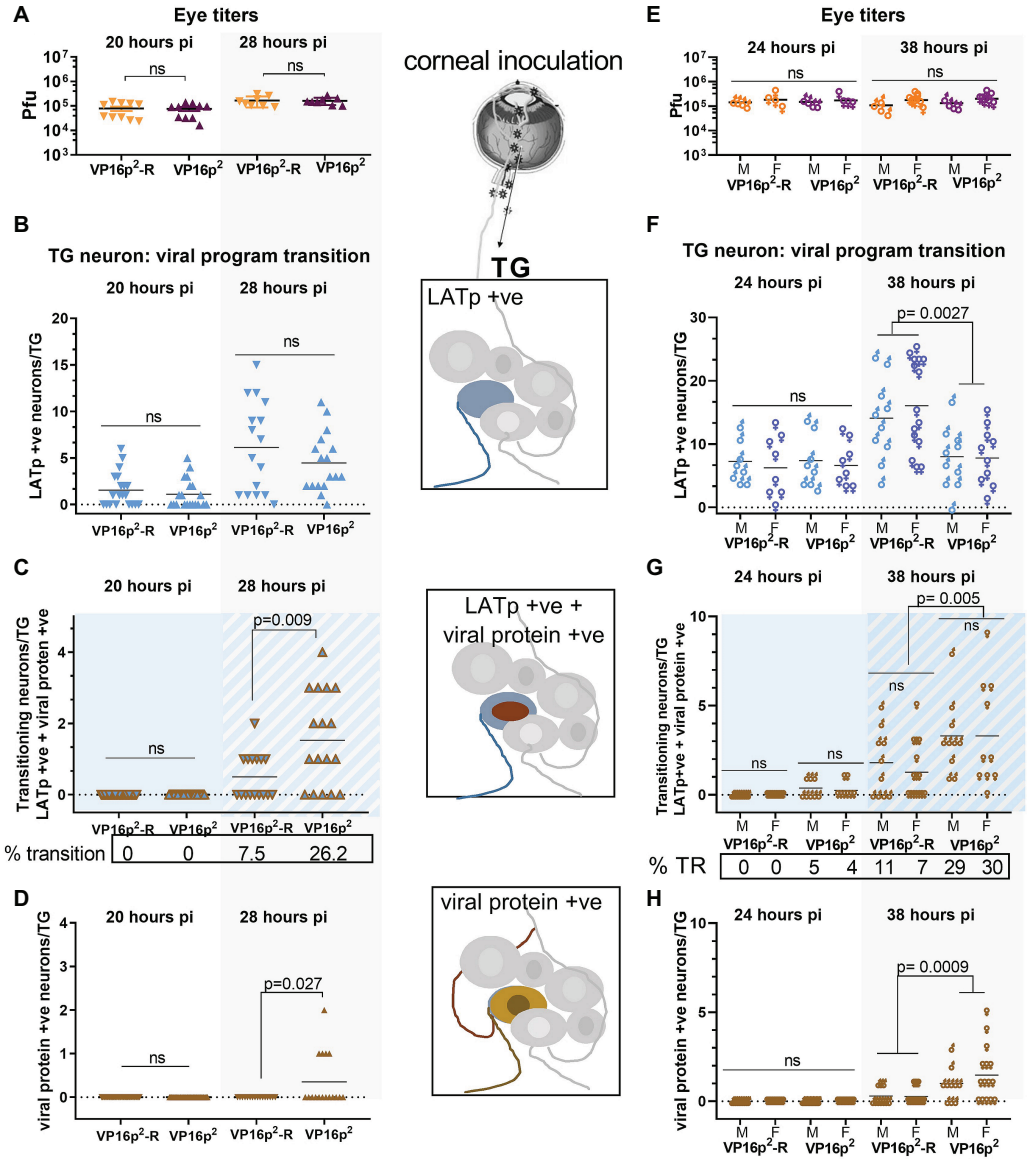
Comparison of default latent state entry and transition into the lytic program regulated by VP16p^2^/5′UTR^2^ or VP16p^1^/5′UTR^1^. Groups of mice were inoculated on scarified corneas with 2 × 10^5^ pfu of 17LpLz/VP16p^2^ or 17LpLzVP16p^2^-R. Eyes were processed (as pairs) for infectious virus titers as detailed in Section “Materials and Methods.” TG was processed for detection of b-gal activity and viral protein expression *in situ* as detailed in Section “Materials and Methods.” The number of marked neurons was enumerated on cleared and pressed whole mounted TG. Panels **(A–D)** include data from male mice. Panels **(E–H)** include data directly comparing male and female mice. The viral titers in eyes at 20 and 28 hpi **(A)** or at 24 and 38 hpi **(E)** were not different among the groups. **(B–D,F,G)** Quantification of viral expression program phenotype in individual neurons within each TG (*n* = 20 at 20 hpi, *n* = 16 at 28 hpi, *n* = 8 at 24 hpi, and *n* = 10–15 at 38 hpi). **(B,F)** Solid blue symbols indicate neurons expressing LATp in the absence of viral proteins. **(C,G)** Blue symbols with brown outline and/or blue background indicate neurons in which expression from both the LATp and viral protein is detected. **(D,H)** Brown symbols indicate neurons in which viral protein alone is detected. Data shown in **(A–D)** are compiled from two biological replicates for each viral mutant at each time point. *n* = 5 for each experiment at 20 hpi and *n* = 4–5 for each experiment at 28 hpi.

Groups of mice were infected on scarified corneas with 2 × 10^5^ pfu of either 17LpLz/VP16p^2^ or 17LpLz/VP16p^2^-R (genomically restored to VP16p^1^/5′UTR^1^). Eyes and TG were harvested from each group at either 20 or 28 h pi. Shown in Figures 8A–D are data from each time point representing results compiled from two independent experiments (no significant differences between the replicate experiments were observed).

Eye titers were not different between 17LpLz/VP16p^2^ and 17LpLz/VP16p^2^-R infected mice at either 20 or 28 hpi (*p* ≥ 0.9, unpaired, two-tailed *t* test) (Figure 8A). The number of blue neurons in the TG at 20 hpi was not different among the groups (*p* = 0.74, unpaired, two-tailed *t* test) (Figure 8B), and no evidence of transition into the lytic program was detected at this time in TG neurons infected with either 17LpLz/VP16p^2^ (0/20) or the genomic rescue of this mutant (VP16p^1^) (0/20) (Figures 8C,D). At 28 hpi, the number of neurons evidencing LATp activity in the absence of viral protein was not different between the mutant and rescue viruses (*p* = 0.30, unpaired, two-tailed *t* test) (Figure 6B). However, there were significantly more neurons expressing combined blue (LATp activity) and viral proteins (transitioning phenotype) in the TG from mice infected with the 17LpLz/VP16p^2^ mutant (*p* = 0.009, unpaired, two-tailed *t* test) (Figure 8C). The number of neurons in which only viral proteins were detected was also significantly more in TG infected with the 17LpLz/VP16p^2^ mutant (*p* = 0.027, unpaired, two-tailed *t* test) (Figure 8D). These data demonstrate that although the initial early default into the latent transcriptional program at 20 hpi is not different between the mutant and its rescue, within 8 h, (i.e., at 28 hpi), 26% of neurons were transitioning into the lytic cycle with the mutant compared to 7.5% with the rescue.

### Transition Into the Lytic Program by HSV-2 VP16 Promoter Targeted Replacement Is Enhanced in Both Male and Female Mice

The preceding transition experiment was performed in male mice. An important question is whether a similar early expression from the LAT promoter followed by a transition into the lytic cycle would also occur in TG neurons during infection in female mice. To test this, age-matched male and female Swiss Webster mice were infected on scarified corneas as above. Eyes and TG were harvested, in this case at 24 and 38 hpi. There were no differences between 17LpLz/VP16p^2^- or 17LpLz/VP16p^2^-R-infected male or female eye titers at either 24 or 38 hpi (Figure 8E). Again at 24 hpi, there were no significant differences in the number of LATp-positive only neurons among the groups (compare Figures 8B,F). While neurons undergoing transition were not observed in either male or female mice infected with the rescue virus at 24 hpi, 4 and 5% of LATp-marked neurons were transitioning into lytic program in males and females infected with 17LpLz/VP16p^2^. By 38 hpi, three-fold more neurons were transitioning in TG infected with 17LpLz/VP16p^2^ compared to the rescue, 29 and 9%, respectively.

### Effect of the Type-1- and -2-Specific VP16 Promoters on Early Spread of Infection in the TG

**Figure 9:**
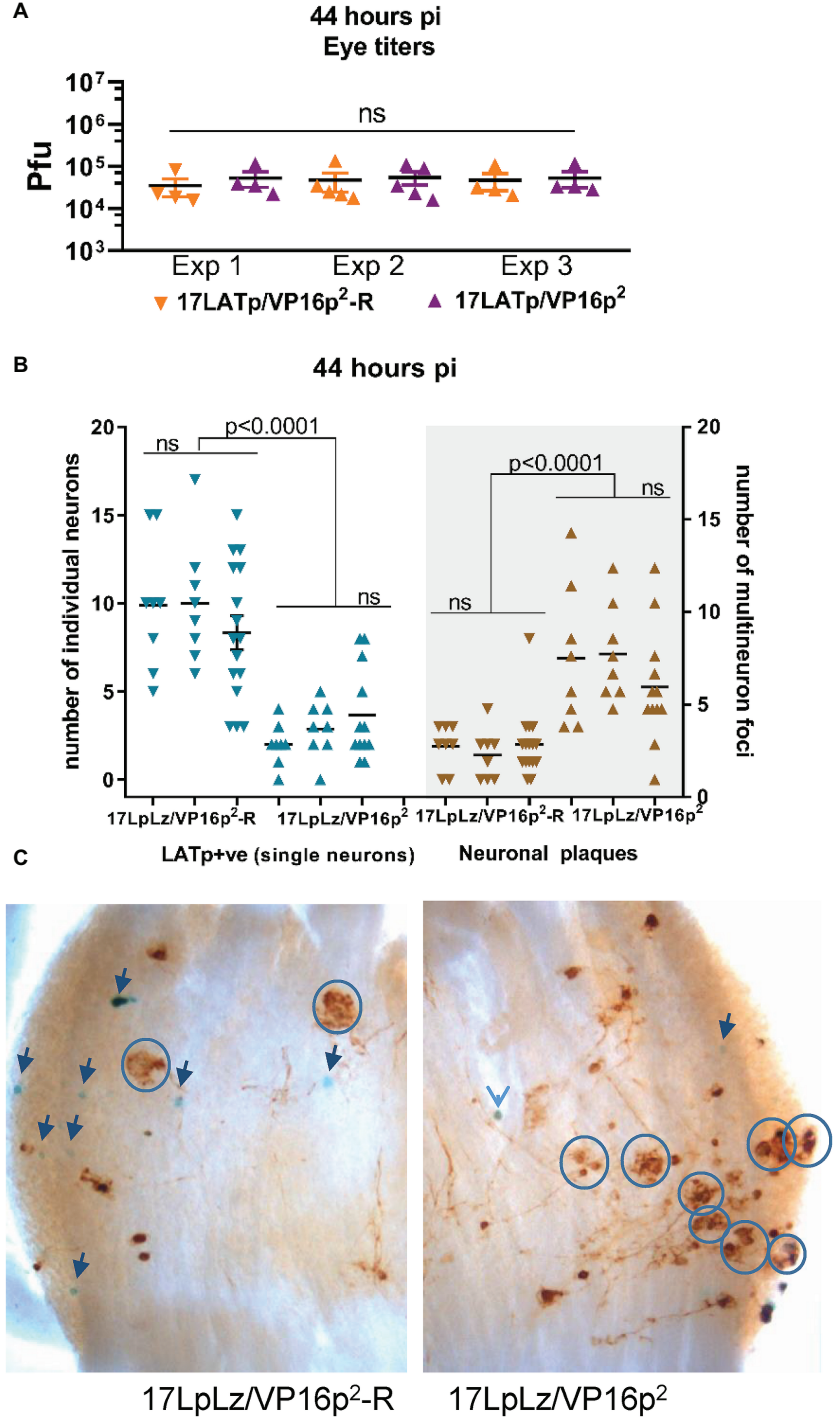
Progression from isolated marked single neurons to multi-neuron (plaque-like) viral protein-positive foci at 44 hpi. Groups of mice were infected with 2 × 10^5^pfu of 17LpLz/VP16p^2^ or 17LpLz/VP16p^2^-R on scarified corneas. At 44 hpi, eyes and TG were harvested. Eyes were processed for infectious virus titers. TG was processed for *in situ* detection of b-gal activity and viral protein expression (see Section “Materials and Methods”). Three independent experiments are shown, Exp 1 and 2, *n* = 8 TG for each group, Exp 3, *n* = 16 and 12 TG for the rescue and mutant, respectively. **(A)** Infectious viral titers in eyes were not different between experiments or between 17LpLz/VP16p^2^- or 17LpLz/VP16p^2^-R-infected mice (ANOVA *p* = 0.97). **(B)** The number of LATp expressing neurons (blue symbols) detected in individual TG are indicated in the left panel. Brown symbols indicate protein expressing multi-neuron foci in each of these same TG (right panel). **(C)** Photomicrographs of representative TG infected with either17LpLz/VP16p^2^-R (left) or 17LpLz/VP16p^2^ (right). Blue circles mark multi-neuron foci, and individual blue neurons are indicated by arrows. The numbers were compared using an ordinary one-way ANOVA with Tukey’s multiple comparison test.

As infection *in vivo* proceeds past ~36 hpi, the viral expression landscape becomes more complex in the TG. Low levels of infectious virus become detectable and virus begins to spread within the TG (Kramer et al., 1998; Sawtell et al., 2006). At 44 hpi, cell to cell spread of infection in the TG is apparent and can be seen in “plaque-like” clusters of viral protein expressing neurons (Figure 9B).

Our hypothesis would predict that once a neuron has transitioned into the lytic cycle and progressed to infectious virus production, that VP16 within the tegument of these virions would be delivered into neighboring cells, be transported into the nucleus and initiate the lytic cycle (paralleling cell to cell spread in tissue culture or corneal infection). The accelerated transition into the lytic cycle from the default latent program linked to the HSV-2 VP16 promoter would be predicted to result in greater numbers of infected multi-cellular foci in the TG. In order to test this, three independent experiments were performed at 44 hpi. Groups of mice were infected as above, and at 44 hpi, eyes and TG were harvested from each group and processed as above. Infectious viral titers in the eyes harvested from 17LpLz/VP16p^2^ and 17LpLz/VP16p^2^-R were not different (Figure 9A). There were no significant differences between the individual biological replicates of the same group with respect to the number of LATp marked neurons or “plaques”. However, the number of “plaques” in 17LpLz/VP16p^2^ compared to its genomic rescue 17LpLzVP16p^2^-R was greater in all three experiments (Figure 7; *p* = 0.0044, *p* = 0.0001, *p* = 0.0025, unpaired, two-tailed *t* test, experiments 1, 2, and 3, respectively). 17LpLz/VP16p^2^-infected TG had nearly four times the number of “plaques” found in 17LpLz/VP16p^2^-R-infected TG at this time pi. Interestingly, there was a reciprocal difference in the number of LATp-positive neurons and the number of plaque-like clusters within TG (Figure 9B). Significantly, as observed at the 38 h time point, fewer LATp-marked neurons were detected in 17LpLz/VP16p^2^ mutant-infected ganglia compared to the VP16p^1^ promoter rescue at this time (*p* < 0.0001 in all three experiments). Photomicrographs of representative ganglia from 17LpLz/VP16p^2^-R (left panel) and 17LpLz/VP16p^2^ (right panel) stained for b-gal activity and viral protein are shown in Figure 9C. Single blue + brown neurons were often surrounded by neurons that appeared to express only viral proteins. This suggested that in contrast to entry into the neuron through the axon, default entry into the latent program is not a feature of infection arising from cell to cell spread directly within the TG.

### Mutation in the Egr1/Sp1 Site in the VP16p^1^/5′UTR^1^ Promoter Influences the Number of Plaque-Like Foci

17VP16πRR was shown previously to exhibit a reduced latent to lytic transition phenotype. In addition, 17VP16p^1^Egr1/Sp1 would be anticipated to display phenotypes similar to those displayed by mutant 17VP16πRR. Groups of mice were infected with 2 × 10^5^ pfu on scarified corneas with 17VP16πRR (*n* = 8), its rescue 17VP16πRR-R (*n* = 8), 17VP16p^1^Egr1/Sp1 (*n* = 8), or its rescue 17VP16p^1^ Egr1/Sp1-R (*n* = 8). At 44 hpi, tissues were harvested, processed, and analyzed as indicated above. TG infected with 17VP16πRR and 17VP16p^1^Egr1/Sp1 contained nearly 10-fold fewer clusters of infected neurons than their corresponding genomic rescues (1.75 ± 1.25 vs. 0.19 ± 0.40, *p* = 0.009) and (2.0 ± 1.86 vs. 0.13 ± 0.34, *p* = 0.0003) for 17VP16πRR and 17VP16pEgr1/Sp1, respectively, and nearly 40-fold fewer than observed with 17LpLz/VP16p^2^ infection.

### VP16p^2^/5′UTR^2^ Promotes Enhanced Response to Reactivation Stress

**Figure 10:**
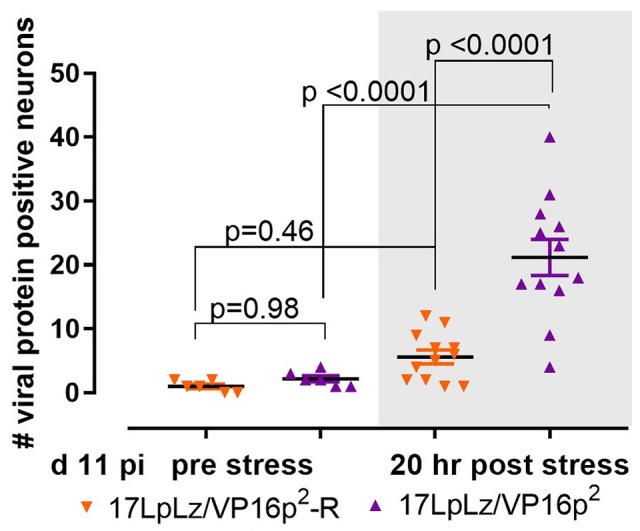
Stress induced transition into the lytic cycle. Mice were infected on scarified corneas with 1 × 10^5^ pfu of either 17LpLz/VP16p^2^ or its rescue17LpLzVP16p^2^-R. At 11 day pi, mice from each infection group were untreated or stressed (see Section “Materials and Methods”). At 20 h post stress, TG from both unstressed and stressed groups from each infection group were dissected and processed for the detection of viral proteins as detailed in Section “Materials and Methods.” The number of neurons expressing viral proteins (evidence of entry into the lytic program) in each TG was counted. Data were analyzed using ordinary one-sided ANOVA with Tukey’s multiple comparison test.

The disposition of the latent viral genome in the TG neuron changes during the course of acute infection (Knipe, 2015; Kristie, 2015; Sloan et al., 2015; Maroui et al., 2016; Nicoll et al., 2016; Cohen et al., 2018). We previously demonstrated that as early as day 9 pi, reactivation stressors promote the transition into the lytic cycle in TG neurons, and we now asked whether the VP16p^2^/5′UTR^2^ is more responsive than VP16p^1^/5UTR^1^. Groups of 30 mice were infected with 1 × 10^5^ pfu of 17LpLz/VP16p^2^ or 17LpLz/VP16p^2^-R (wt HSV-1 genotype) on scarified corneas. On day 11 pi, surviving mice were randomized into two groups for each virus and half were subjected to stress. TG was excised and whole ganglia were stained for viral proteins, cleared, mounted and examined as described in Section “Materials and Methods.” Few neurons were positive for viral proteins in TG from the untreated groups regardless of whether VP16 was expressed from type-1 or type-2 regulatory sequences and the number found was not different between groups (*p* = 0.98, ordinary one-way ANOVA with Tukey’s *post hoc* analysis, Figure 10). The numbers of positive neurons in TG from animals at 20 h post stress increased in both groups. This increase was not significant in mice infected with the wt genomically restored virus (*p* = 0.46), but a significant increase in positive neurons was observed in TG of mice infected with the mutant expressing VP16 from the HSV-2 promoter/5′UTR^2^ (*p* < 0.0001) (Figure 10). We conclude that the VP16p^2^/5′UTR^2^ regulatory sequences were significantly more responsive to a stress that induces reactivation from the “early” latent state than the HSV-1 sequences.

## Discussion

**Figure 11:**
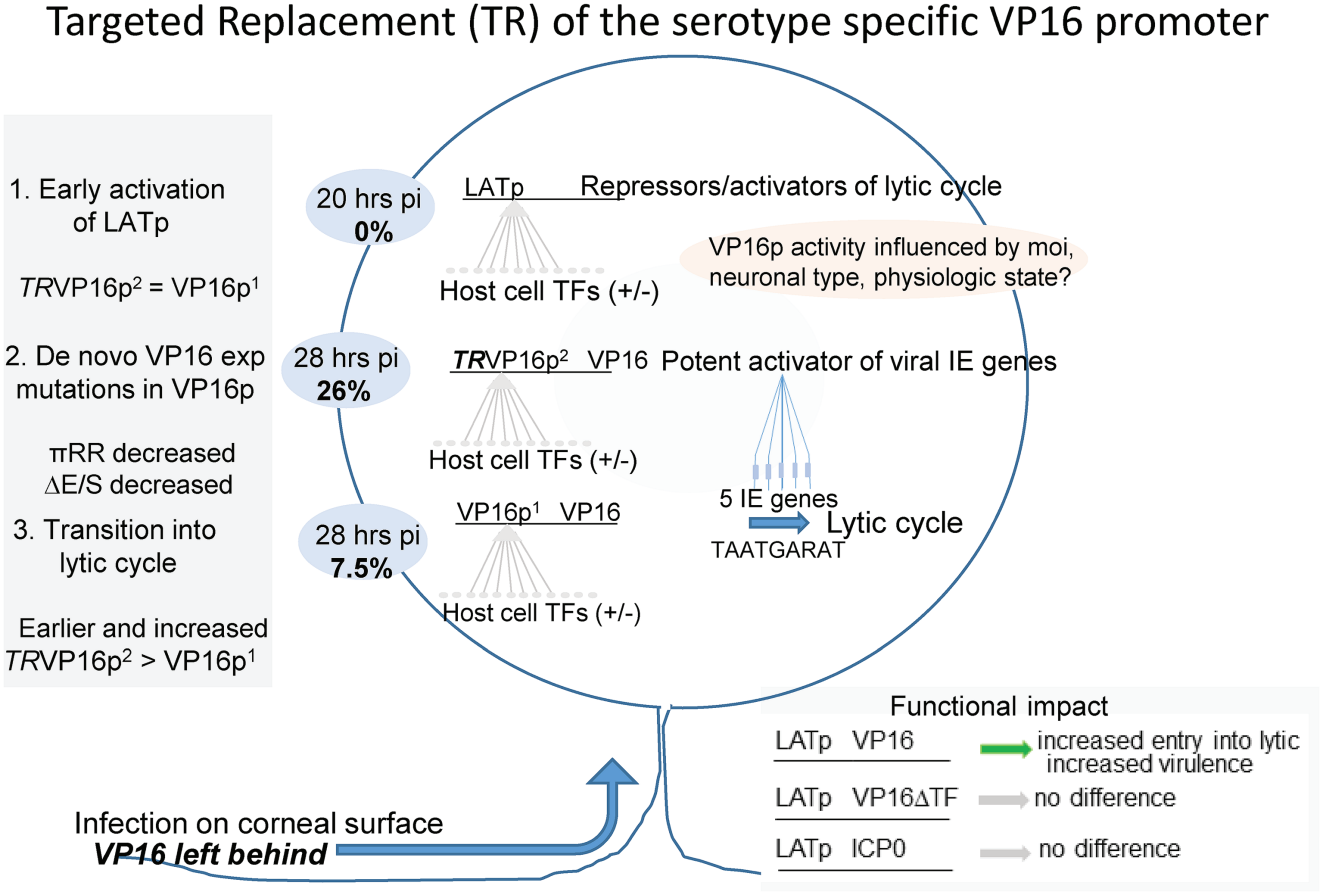
Summary of the role of VP16 and its regulation in the earliest stages of HSV infection of sensory neurons *in vivo*. Sensory neurons residing in the trigeminal ganglion become infected with HSV through their axonal endings distributed at the body surface site of infection. This unique mode of entry into the neuron has long been thought to play a central role in the outcome of infection in the infected neuron. Early studies supported the concept that the outcome was binary-entering either latency in which only the LAT gene is abundantly transcribed, or lytic infection resulting in virus production and neuronal death (Margolis et al., 1992, 2007; Sawtell and Thompson, 1992a,b; Lachmann and Efstathiou, 1997). However, approaches that allow visualization and quantification of very rare events in the TG have revealed that individual neurons enter the latent program first, and a portion of these transition into the lytic cycle. This transition requires the *de novo* expression of VP16 (Thompson et al., 2009; Sawtell and Thompson, 2016a,b). This is important because it identifies a regulatory nexus residing in the VP16 gene, with the potential for multiple neuronal context dependent inputs feeding into the VP16 regulatory machinery. We propose that the VP16 promoter region functions as a host cell micro-environment sensor, which in turn is modified by systemic inputs. This regulation of VP16 expression serves to maximize the establishment of latency while minimizing severe morbidity or mortality in diverse hosts infected under differing conditions (age, immune system status etc.) with HSV-1 strains that vary in their pathobiologic properties. The near ubiquity of the virus in the human population at large and relative rarity of serious sequelae of infection is testimony to the success of this evolutionary strategy. (1) Latency is favored in neurons because tegument proteins including VP16 are not transported through axons to neuronal nuclei along with the viral genome. Because the IE genes are not transactivated and ICP4 protein (a repressor of the LAT promoter, LATp) is absent, the LATp interacts with a variety of host TFs, is turned on, and expresses RNAs that repress and/or possibly enhance lytic infection (Watson et al., 2018). (2) Embedded in both the HSV-1 and HSV-2 VP16 regulatory regions are sequences that promote its *de novo* expression in some of these neurons that are already expressing the LATp. (3) *De novo* VP16 expression initiates the lytic cascade in these neurons. By 28 hpi, 26% of the LATp+ neurons show evidence of viral protein expression when VP16 is driven by the type 2 promoter in the context of HSV-1, whereas about 7.5% are positive in wt HSV-1. This initiation of lytic gene expression requires functional VP16 transactivation and does not occur if ICP0 instead of VP16 is expressed during the default latent state (Thompson et al., 2009).

A schematic summary is shown in Figure 11. Evidence from diverse studies support the idea that the tegument protein VP16, which initiates lytic infection at low moi by transactivating the viral immediate early genes (Campbell et al., 1984; Bzik and Preston, 1986; Ace et al., 1988; Wysocka and Herr, 2003; Thompson et al., 2009; Sawtell and Thompson, 2016a,b), is not transported through axons efficiently favoring latency (Thompson et al., 2009; Antinone and Smith, 2010; Aggarwal et al., 2012; Hafezi et al., 2012; Smith, 2012; Koyuncu et al., 2015, 2017; Sawtell and Thompson, 2016a,b). It is noted that this acute stage “default” latent transcription program is unlikely to share all features of a fully consolidated latent infection. The latter requires extensive changes to the chromatin structure on the viral genome which occurs over a prolonged period of time and involves interactions with subnuclear structures (Knipe and Cliffe, 2008; Cliffe et al., 2009, 2015; Kwiatkowski et al., 2009; Bloom et al., 2010; Lu and Triezenberg, 2010; Roizman et al., 2011; Knipe, 2015; Kristie, 2015; Sloan et al., 2015; Maroui et al., 2016; Nicoll et al., 2016; Cohen et al., 2018). Importantly, productive infection in TG neurons also occurs in animal models and humans, and this acute stage viral replication in the peripheral nervous system is important to maximize the latent burden and optimize reactivation potential in experimental models (Thompson and Sawtell, 2000). We and others are investigating how this viral replication is initiated in TG neurons. There is evidence for several mechanisms that might initiate HSV replication in neurons including alternate transport mechanisms for VP16 through axons (Antinone and Smith, 2010), high moi neuronal infections that obviate the requirement for VP16 (Koyuncu et al., 2015) as is well documented in cultured cells (Ace et al., 1989), a binary choice made based on neuronal subtype (Yang et al., 2000; Margolis et al., 2007; Bertke et al., 2013) and an unexpected neuronally directed expression program embedded in the HSV-1 VP16 promoter/5′ UTR that appears to induce the synthesis of VP16 protein *de novo* (Thompson et al., 2009; Sawtell and Thompson, 2016a,b).

This latter hypothesis is supported by several related observations. Infection of mice *via* the cornea results in detectable expression of only the latency associated transcript promoter (LATp) at very early times (18–32 hpi). Expression of lytic phase promoters of the immediate early and leaky late kinetic classes and their cognate proteins occurs about 12–14 h after the LATp is expressed, and only in cells already “marked” by LATp-driven *E. coli* beta-galactosidase (b-gal) expression (Thompson et al., 2009; Sawtell and Thompson, 2016a,b and see also Figures 8, 9). The LAT promoter is strongly repressed by the immediate early protein ICP4 in cells including neurons during acute infection (Batchelor and O’Hare, 1990; Margolis et al., 1992; Sawtell and Thompson, 1992a,b; Farrell et al., 1994); we can therefore conclude that this transition out of the default latent transcription program requires *de novo* lytic protein expression that occurs after the LATp is activated. That the transition out of the latent state is driven by *de novo* VP16 expression is supported by several additional observations. (1) Analysis of a VP16 promoter/b-gal reporter mutant in the background of the VP16 transactivation-deficient mutant in1814 (Ace et al., 1989) demonstrated that the VP16 promoter can be activated in infected neurons in the absence of other viral proteins (Thompson et al., 2009). (2) Expression of an extra copy of VP16, but not of ICP0, *de novo* from the LATp eliminates the default latent state. With this mutant, virus replication in TG occurs within 20 hpi, is more extensive in TG and brain, and virulence is significantly enhanced (Sawtell and Thompson, 2016a,b). Mutation of TF binding sites downstream of the VP16 TATA resulted in replication reduced about 100-fold in TG and the exit from the default latent pathway into the lytic pathway was significantly reduced, as was virulence (Sawtell and Thompson, 2016a,b). Yet these mutations had no effect on the kinetics of VP16 expression or viral replication in cells in culture or on replication on the mouse eye *in vivo* (Sawtell and Thompson, 2016a,b). This is consistent with early studies mapping TF sites important for normal leaky late VP16 expression to regions upstream of the TATA box (Lieu and Wagner, 2000).

In this study, we first refined the analysis of sequences required for the *de novo* expression of HSV-1 in neurons. Introducing a four base change in the core matrix of a canonical Egr-1/Sp1 site located in the HSV-1 VP16 5′UTR resulted in mutants that replicated like wt in cultured cells and mouse eyes (Figures 2A, 5B). This later finding is important because the viral load entering the TG from the eye at early times was therefore equivalent as confirmed by QPCR for the viral genome in TG. Despite this, viral replication in TG was reduced by two orders of magnitude (Figure 4C), and virulence was abrogated. We emphasize that we do not yet know how these sequences function, but favor the hypothesis that this region represents a combinatorial TF-binding site that can confer both positive and negative regulation to VP16 transcription in neurons because (1) both Egr-1 and Sp1 can bind to the site with wt affinities (Figure 4); (2) many host genes including stress responsive neuronal genes are reciprocally regulated by these factors in response to stress (Carrasco-Serrano et al., 1998; Nitsch et al., 1998; Contestabile, 2008; Liu et al., 2014; Wu et al., 2017); and (3) the stress induced factor Egr-1, which can displace the ubiquitous positive factor Sp1, induces its corepressor NAB2 that rapidly induces a silenced chromatin state (Houston et al., 2001; Kumbrink et al., 2005). This latter feature is compatible with the fact that the vast majority of latent viral genomes do not respond to systemic stressors by exiting latency (Sawtell and Thompson, 1992a,b, 2016a,b; Sawtell, 2005; Thompson and Sawtell, 2006; Thompson et al., 2009). We note, however, this site can bind other TFs and is also located in the 5′UTR of the VP16 mRNA. 5′UTR sequences can influence protein production in many ways exclusive of transcriptional regulation. Unfortunately, it is currently not possible to computationally predict these functional aspects of 5′UTRs in eukaryotes (reviewed in Leppek et al., 2018), but most experimentally determined sequences act to inhibit protein expression (reviewed in Rhodes and Lipps, 2015). For example, the region containing the HSV-1 overlapping Egr-1/Sp1 site is predicted to have the capacity to form a single DNA as well as an RNA G-Quadruplex structure (Kikin et al., 2006). This structure is known to inhibit ribosome scanning on mRNA and reduce protein expression (Rhodes and Lipps, 2015). The 4-bp mutations would be expected to eliminate the predicted G-Quadruplex structure (Kikin et al., 2006), and if this was a functional 5′UTR RNA G-Quadruplex, the mutations would be expected to increase, rather than decrease VP16 protein production.

In order to extend our analyses of VP16 regulation and potentially avoid the problem of mutation of 5′ UTR sequences, we took advantage of the existence of a second natural human HSV VP16 promoter, that of HSV-2. We asked whether the HSV-2 promoter/5′UTR, which has a very different structure, could also direct the *de novo* VP16 synthesis *in vivo* in the context of the HSV-1 genome. HSV-1 mutants in which the HSV-1 VP16 promoter and 5′UTR were replaced with those of HSV-2 leaving all other genes and all ORFs as HSV-1 were produced in the background of 17LATpLacZ (which labels neurons expressing the latent transcription program with b-gal). In this way, the effects of the HSV-2 VP16 regulatory region on latent/lytic transition were isolated from all other HSV type-specific differences. If the hypothesis is correct, despite its very different architecture we would expect to see that the HSV-2 promoter would induce the transition of HSV-1 from the default latent program into the lytic program. This may be a unique feature of the VP16 promoters, as another leaky late promoter/5′UTR employed to drive VP16 (that of VP5) fully supports viral replication in culture and mouse eyes, but fails to efficiently transition out of the default latent state (Thompson et al., 2009). Furthermore, we anticipated that due to the presence of 13 tandem direct repeats that contain multiple putative overlapping canonical Egr-1/Sp1 sites in the HSV-2 promoter, this promoter might exhibit altered kinetics for the transition into lytic infection from default latency.

The targeted replacement mutant displayed phenotypes consistent with these hypotheses. The type-2 sequences efficiently directed the exit from the default latent state and initiated productive viral replication in TG neurons. Indeed, earlier and more extensive disruption of the latent transcription program and entry into the lytic program was evident by 28 hpi (Figure 8), this increase in transition continued through time (Figure 9), increased and prolonged viral replication was observed in the CNS (Figure 6), and a significant increase in virulence resulted in reduced mouse survival (Figure 7). The type-2 promoter mutants also displayed increased responsiveness to systemic stresses that induce reactivation *in vivo* (Figure 10).

The phenotypes displayed by the type-2 promoter mutants are robust, and our transition index and pathobiological assays are sensitive enough to permit us to determine the relative roles played by the 13 tandem Erg-1/Sp1 sites and/or other regulatory regions in *de novo* VP16 expression in both the HSV-1 and HSV-2 backgrounds. One advantage is that the putative Egr-1/Sp1 sites in HSV-2 are in the distal promoter and mutations will not affect mRNA structures. Such studies may help illuminate the signaling pathways that lead to acute viral replication in the PNS, as well as reactivation. This knowledge may help direct the design of safer live vaccine candidates or reveal potential targets for pharmacological interventions.

## Data Availability

All datasets generated for this study are included in the manuscript.

## Ethics Statement

This study was carried out in accordance with the recommendations of the Guide for the Care and Use of Laboratory Animals. The protocol was approved by the Children’s Hospital Institutional Animal Care and Use Committee (protocol# IACUC2017-0081).

## Author Contributions

Both authors contributed equally to the ideas underlying the investigation, the experimental design, execution, interpretation of the data, and preparation of the manuscript.

### Conflict of Interest Statement

The authors declare that the research was conducted in the absence of any commercial or financial relationships that could be construed as a potential conflict of interest.
